# Idiopathic Retroperitoneal Fibrosis: An East African Diagnostic Challenge

**DOI:** 10.7759/cureus.15683

**Published:** 2021-06-16

**Authors:** Sayed K Ali, Katie Du, Kelvin Orare

**Affiliations:** 1 Medicine, Aga Khan University Hospital, Nairobi, KEN; 2 Internal Medicine, Aga Khan University Hospital, Nairobi, KEN

**Keywords:** retroperitoneal fibrosis, diagnosis and treatment, east africa, kenya, steroids

## Abstract

Retroperitoneal fibrosis (RPF) is a rare disease with variable etiology characterized by chronic inflammation and fibrosis of tissue surrounding the abdominal aorta and iliac arteries. The majority of cases of RPF remain idiopathic and mostly reported in men. Glucocorticoids remain key in the treatment of RPF. Little has been reported on RPF in sub-Saharan Africa.

## Introduction

Retroperitoneal fibrosis (RPF) is a rare disease with variable etiology characterized by chronic inflammation and fibrosis of tissue surrounding the abdominal aorta and iliac arteries, often leading to other complications such as aortic aneurysms and ureteral obstruction [1]. Approximately two-thirds of RPF cases are idiopathic [1-5]. Men are two to three times more likely to develop RPF than women with a mean onset of around 55-60 years [3-4]. CT and MRI remain crucial in making a diagnosis of RPF and can often be aided by biopsy of the affected region [1,3]. Steroids, and steroid-sparing immunosuppressants, remain the main treatment modalities for RPF [1].

## Case presentation

A 39-year-old man presented to our institution with abdominal pain, intermittent episodes of low-grade fever, yellowness of his eyes, and loss of appetite for two weeks. In addition to intermittent itching, he also reported a four-kilogram weight loss over the course of six months. He denied any dysuria, hematuria, skin lesions, or neurological deficits.

His past medical history was significant for an appendectomy as a child. He denied active use of alcohol or tobacco. He worked as an administrator for a non-governmental organization and was able to perform all his activities of daily living. His father and mother were alive and doing well, with no significant health issues. He denied using prescribed, herbal, or over-the-counter medications. He denied any significant travel history outside his work area.

On examination, he was cachectic, with mild pallor and jaundice. He had a blood pressure of 118/74 mmHg, pulse rate of 108 beats per minute, and temperature of 36.6°C. He had bilateral inguinal lymphadenopathy with skin excoriation marks. His left testicle was tender on palpation, but no urethral discharge or penile lesions were appreciated. His abdomen was scaphoid, with a mildly enlarged tender liver. A Lanz incision scar was present. He had no stigmata of chronic liver disease. His lower extremities appeared normal with no edema.

No neurological deficits were appreciated on his exam and his gait was normal.

The routine laboratory tests performed revealed an elevated white cell count of 16 x 106/L and hemoglobin of 8.4 g/dL (normocytic, normochromic with normal mean corpuscular volume [MCV]). His initial ferritin measured 1512 ng/mL, serum iron of 5.24 umol/L, and transferrin of 1.84 g/L suggestive of anemia of inflammation. His inflammatory markers were elevated with an erythrocyte sedimentation rate (ESR) of 55 mm/Hr, and a C-reactive protein (CRP) of 171 mg/dL. Liver function test revealed an elevated alkaline phosphatase of 408 U/L, gamma-glutamyltransferase (GGT) of 281 U/L, and direct hyperbilirubinemia of 103 umol/L with normal transaminases. His electrolytes including his creatinine were normal.

Hepatitis A, B, and C serology were negative. Blood cultures and urine cultures were also reported as negative. A rheumatoid factor was positive with negative anti-citrullinated peptide (anti-CCP), anti-nuclear, anti-smooth muscle, anti-mitochondrial and antineutrophil antibodies (C and P). A liver autoimmune panel was also negative. Complement and immunoglobulin levels were within normal ranges. Serum electrophoresis was negative for monoclonal gammopathy. Lactate dehydrogenase (LDH) and IgG4 subclass antibodies were within normal limits. We did not perform a serum QuantiFERON.

**Figure 1:**
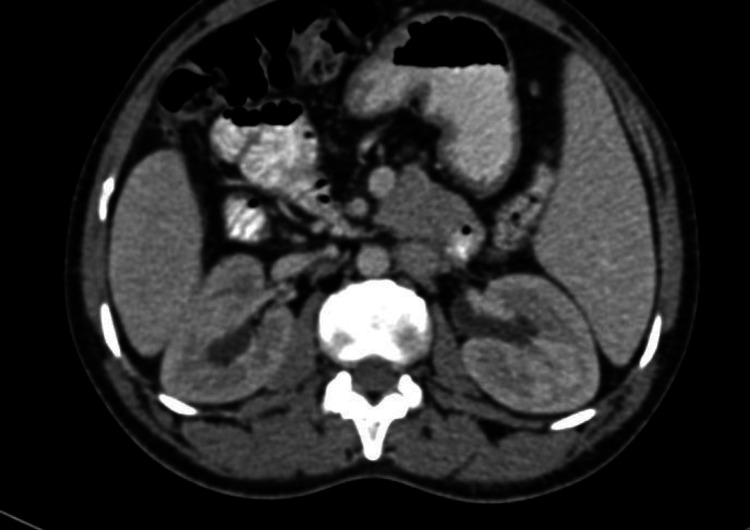
Axial enhanced CT showing mild periureteric fibrotic changes and mild hydronephrosis.

A testicular ultrasound done was reported as normal with incidental finding of right inguinal lymphadenopathy. An abdominal CT scan showed mild splenomegaly with mild bilateral hydroureteronephrosis with retroperitoneal fat stranding suggestive of early retroperitoneal fibrosis (Figure 1). An ultrasound of the liver showed a gall bladder that was of normal size and wall thickness, no dilatation of the intrahepatic bile ducts, and no calculi. A follow-up FibroScan showed no fibrosis. An MRI of the liver was also reported as normal. A liver biopsy showed mild non-alcoholic hepatitis, but no fibrosis.

**Figure 2:**
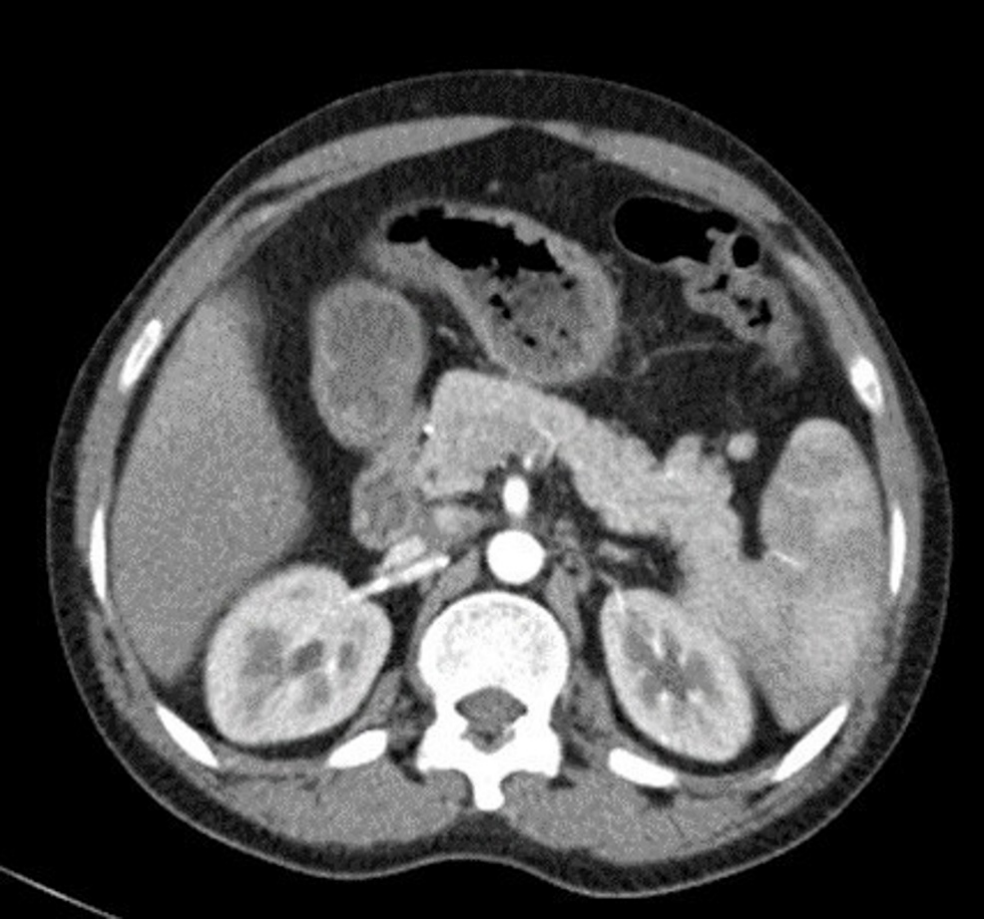
Axial enhanced CT showing resolution of the periureteric fibrotic changes and mild hydronephrosis.

A diagnosis of idiopathic RPF was made based on his clinical and radiological findings. He was started on treatment with corticosteroids (prednisone 60 mg/day) with marked improvement in his symptoms within 72 hours. Corticosteroids were gradually tapered off over a six-month period. Follow-up laboratory testing done at two months showed decreasing inflammatory markers (CRP 55 mg/dL and ESR 29 mm/Hr) and resolving cholestasis (alkaline phosphatase [ALP] 127 U/L and GGT 100). At six months, a repeat CT scan showed resolution of the hydroureteronephrosis and the RPF (Figure 2), and his liver function and hemoglobin had returned to baseline.

## Discussion

RPF is a rare disease with variable etiology characterized by chronic inflammation and fibrosis of tissue surrounding the abdominal aorta and iliac arteries, often leading to compression of other abdominal structures and blockage of ureters [1]. Although secondary forms of RPF precipitated by underlying causes have been reported, approximately two-thirds of RPF cases are idiopathic [1-5]. The true epidemiological parameters of RPF are unknown, however, it is estimated to be one per 200,000 to 500,000 persons per year [3]. Men are two to three times more likely to develop RPF than women [3-4]. The mean age of onset ranges from 45.8 to 69.4 years [6].

RPF diagnosis is complicated by non-specific initial symptoms, such as lower back and abdominal pain, and discomfort in the groin area [1,3,6-7]. Systemic symptoms, including fatigue, weight loss, and fever, can precede or coexist with the aforementioned localized manifestations [1,4-6,8]. A prolonged delay between symptom onset and correct diagnosis can lead to an increased likelihood of severe complications such as renal failure [1,9-10]. In fact, since there is no specific laboratory test for RPF, it is commonly diagnosed alongside acute kidney failure [10].

CT scan and MRI are key to making RPF diagnosis [1,5,8,11-12]. The typical imaging finding of idiopathic RPF is an irregular retroperitoneal soft-tissue mass extending around the abdominal aorta, iliac artery, inferior vena cava, and ureters [1,5,8,10]. MRI has the additional advantage of enhanced soft-tissue contrast, while the fibrous plaques are isodense to muscle on non-contrast-enhanced CT [1,11-12].

However, since patients with RPF may be experiencing renal failure, special consideration must be taken into account when performing contrast-enhanced CT as a diagnostic method [12]. Neither CT nor MRI can be used as the sole basis for differentiating between benign and malignant RPF [10].

Diagnosis of RPF relies primarily on imaging, however additional, supplemental methods, such as biopsies, may be useful when there is a lack of expertise with disease management, presence of underlying medical conditions, atypical localization of the mass (e.g., periureteral, perirenal), or resistance to therapy [10]. In our patient, it was felt that the technical risk of a biopsy outweighed the benefit and hence this was not pursued.

Intravenous urography and ultrasound may also be used to aid initial diagnosis [1,11,13]. Since RPF histology is poorly characterized, a positive histological diagnosis is not considered necessary in order to start treatment [10]. In our patient, due to his non-specific symptoms and multiple physical findings, including an association of RPF with various autoimmune and fibro-inflammatory conditions [5], we carried out a wide range of tests to further investigate a broad differential diagnosis.

Laboratory results, such as CRP and ESR inflammatory marker levels, are usually used to monitor disease progression and response to treatment [1,8].

Currently, research suggests there may be an association between tuberculosis and RPF [14]. A case study from Italy reported the simultaneous diagnosis of extrapulmonary tuberculosis and idiopathic RPF [14]. The diagnosis of RPF was idiopathic and not secondary because the fibrous mass did not cause displacement of the aorta [14]. After the patient was treated with antituberculosis medication, both the mediastinal adenopathy and RPF regressed [14]. This case suggests a causal relationship between tuberculosis and RPF that may be of special interest to sub-Saharan medical research as tuberculosis is one of the top causes of illness and death in Africa [15]. Even though tuberculosis was entertained as a possible diagnosis in our patient, his rapid improvement on steroids deflected from this diagnosis.

Early-stage RPF is usually treated with glucocorticoids, however, if there are contraindications, or if patients develop resistance, or experience steroid-related toxicity, tamoxifen [1,5,7-8,13,16] and immunosuppressants are possible alternatives [1,4-5,9,13]. Tamoxifen is a nonsteroidal anti-estrogen, first used in the treatment of RPF in 1991 [16], that reverses the growth of fibrous plaques. The mechanisms of action of immunosuppressants are unclear [5], however, mycophenolate mofetil [1,4-5,8-9] and cyclophosphamide [1,4-6,8-9,13] have been shown to be effective. If neither tamoxifen nor immunosuppressants are effective, rituximab [5,17], a B-cell depleting agent, and tocilizumab [5,18], the anti-Il-6 receptor monoclonal antibody, are possible lines of therapy. Methotrexate [1,5-6,8-9,19-20], an antimetabolite, is a first-line therapy choice in cases of relapsing RPF. Ureteral stenting [1,4-6,8,13] or nephrostomy can be used to relieve urinary obstructions [1,4-6,8,13,20].

## Conclusions

Although RPF is a rare disease, research in its diagnosis and management has significant clinical implications. The lack of standardized protocols and parameters highlights the importance of approaching each potential case with a combination of an accurate history, a good physical exam, and appropriate diagnostic tools. Some of these diagnostic modalities might be limited in a resource-limited setting, limiting the ability to make an accurate diagnosis. In addition, patient education on this rare condition remains key to ensure compliance with medications and follow-up.
